# Personal identity, possible worlds, and medical ethics

**DOI:** 10.1007/s11019-022-10089-w

**Published:** 2022-05-26

**Authors:** Nils-Frederic Wagner

**Affiliations:** Institute for the History, Theory, and Ethics of Medicine, University of Mainz Medical Center, Am Pulverturm 13, 55131 Mainz, Germany

**Keywords:** Personal identity, Thought experiments, Intuitions, Psychological continuity, Animalism, Medical ethics

## Abstract

Thought experiments that concoct bizarre possible world modalities are standard fare in debates on personal identity. Appealing to intuitions raised by such evocations is often taken to settle differences between conflicting theoretical views that, albeit, have practical implications for ethical controversies of personal identity in health care. Employing thought experiments that way is inadequate, I argue, since personhood is intrinsically linked to constraining facts about the actual world. I defend a moderate modal skepticism according to which intuiting across conceptually incongruent worlds constitutes ‘invalid intuition-inferences’—i.e., carrying over intuitions gathered from facts about possible worlds that are at odds with facts about the actual world, for the purpose of making claims about real-life persons and their identity, leads to conceptual incongruences. Such a methodological fallout precludes accurate, informative judgments about personal identity in the actual world, calling into question the adequacy of thought experimental considerations for potential real-world applications in medical ethics.

## Introduction

Controversies about personal identity figure prominently in wide-ranging ethical issues in health care, such as abortion (McInerney 1990; Warren 1977; Oderberg 1997); advance directives, in particular with regard to neurodegeneration (Buchanan 1988; DeGrazia 1999; Vollmann 2001; Limbaugh 2016); and Deep Brain Stimulation (DBS) (Lipsman and Glannon 2013; Nyholm and O’Neill 2016; Müller et al. 2017). Employed as therapeutic interventions for neurodegenerative diseases, DBS bears the potential to significantly alter patients’ psychological make-up. As such, DBS can have an impact on the ontological, moral, and legal status of patients undergoing such treatment. In the case of advance directives, patients suffering from neurodegenerative diseases such as Alzheimer’s must make a call on behalf of their future selves, that, however, might no longer be identical to the ‘original’ self that has signed the advance directive. The moral permissibly of abortion is closely linked to the moral status of fetuses and the persons they potentially become: if a fetus is considered a person in the making, abortion would immorally deprive it of a ‘future like ours’, or so goes Marquis’s (1989) seminal argument.

These and related discussions about potential identity disruptions in medical ethics are often implicitly (occasionally explicitly) based on philosophical theories of personal identity. Theories that are, in turn, frequently defended by appeal to counterfactual thought experiments that, despite being logically possible, are at odds with the set of facts of the actual world that affect real-persons and their identity. These kind of thought experiments, as I’ll argue, have no bearing on real-world cases, and should be taken with a grain of salt. Before turning to argue why this is so, some conceptual tidying up is in order.

The focal point of theorizing about personal identity has been to sort out two related questions:

1. what are synchronic conditions of personhood, and
2. how do persons, so defined, persist through time?

Not all and only human beings are persons. The concept of personhood in principle allows for non-human persons, as well as artificial and alien persons. But human persons are the solely uncontested case to date, and thus inform our theorizing about personhood and personal identity. There is, then, reason to start with the constitution of human persons as the paradigm. Accordingly, many recent attempts to answer (1) are based on what I shall call Orthodox Commitments about personhood.^1^

Realism: ‘Person’ is a natural kind, picking out creatures that de facto exist in the actual world.

Naturalism: Persons are biological beings whose existence is a matter of empirical facts.

Cognitivism: Persons are equipped with higher-order cognition that enables diachronic self-consciousness.

In a nutshell, persons as we know them, are real, biological beings that, via higher-order cognition, can first-personally conceive of themselves as themselves persisting over time (hereafter, simply ‘real-life persons’).

Orthodox Commitments entail that personhood is intrinsically linked to, and constrained by, facts about the actual world. This is because these constraining facts have crucially informed the conceptual genesis of personhood, and continue to govern its practical application. Had the actual world been different, allowing, say, for people splitting into two equally adequate successors, the concept of personhood, too, would have evolved differently. Questions of, for example, divided identity that don’t crop up as things stand (apart from thought-experimental worries), would have arisen as legitimate concerns in a world where people do split. Call the intimate relation between personhood and the actual world’s constraining facts Intrinsic Linkage.

Even though Orthodox Commitments appear both independently plausible and are widely agreed upon, philosophers that share these commitments often disagree as to their entailment for answering (2). For brevity’s sake, I focus on two main contenders:

Friends of Psychological Continuity theories hold that, necessarily, person *x* at t_1_ is identical to person *y* at t_2_ if and only if *x* and *y* are psychologically continuous (e.g., connected via perpetual chains of psychological continuity).

Friends of Animalism hold that, necessarily, person *x* at t_1_ is identical to person *y* at t_2_ if and only if *x* and *y* are biologically continuous (e.g., connected via perpetual chains of biological continuity).

How psychological and biological continuity is spelled out precisely differs between various proponents of these views. For my purposes, it shall suffice, though, to have a general overview in place.

Here’s how I proceed: in section two, I discuss reasons why thought experiments loom large in settling differences between rival views of personal identity. In so doing, I distinguish between hypothetical thought experiments that are in keeping with facts about the actual world relevant to persons and their identity, and counterfactual thought experiments that are at odds with these facts. In section three, I survey cerebrum transplant thought experiments as a case in point. In section four, I argue that counterfactuals of such nature are inadequate to settle differences between rival views of personal identity. The reason for this is that intuiting from facts about possible worlds, where these facts violate those of the actual world, to then reapply these intuitions to the identity of real-life persons, constitutes invalid intuition-inferences. Because of Intrinsic Linkage, such intuition-inferences lead to conceptual incongruences across worlds and, thus, cannot generate accurate, informative judgments about personal identity in the actual world. In section five, I argue, furthermore, that such invalid intuition-inferences are at odds with Orthodox Commitments, as they conflate de re necessity and de re possibility about persons. In section six, I look at the impact that the rise of experimental methods in philosophical debates on personal identity has recently had on real-life cases in medical ethics. In so doing, I argue for a more balanced approach of both theory informing the practical approach clinical ethicists take towards real-life cases, as well as real-life cases inform theorizing about personal identity. In section seven, I take stock.

## Thought-experimenting in personal identity

Differences between rival views of personal identity often appear most strikingly in thought experiments that frequently concoct bizarre counterfactual propositions. Derek Parfit’s early work on personal identity has played a pivotal role in reigniting this way of thought-experimenting. Yet, Parfit tells us that “different views about personal identity make different claims about actual people, and ordinary lives” (Parfit 1984). So, thought experiments about possible worlds are not mere illustrations of theories and their implications. Nor ends in themselves. Rather, they are introduced as adequate, genuine attempts to sharpen our conceptual understanding of personhood and personal identity regarding real-life persons.

Along these lines, Parfit offers a reason why thought experiments about possible world modalities are evoked, claiming that “the difference between these views is clearer when we consider certain imaginary cases. Most of the arguments appeal, in part, to such cases. It may be impossible for some of these cases to occur, whatever progress may be made in science and technology” (ibid.).^2^ Ever since, there has been little departure from such liberal application of thought experiments that are profoundly at odds with the facts about the actual world relevant to persons and their identity.^3^ It is, for example, common currency for advocates of Psychological Continuity theories and Animalism alike to employ thought experiments to showcase how their views differ. And, more importantly, to elicit intuitions that allegedly pull the uninitiated towards their respective view. The primary reason for employing thought experiments is, then, to carve out what is conceptually essential about persons and their identity by isolating core conceptual features. According to the prevailing view, doing so requires imagining away ontologically insignificant contingencies about the world that real-life persons happen to inhabit.

What follows is not a critique of thought experiments that aim at imaging away ontologically insignificant contingencies about persons *per se*; let alone critiquing thought experiments in general. Rather, I take issue with the widespread tendency to employ thought experiments that evoke possible world modalities to make inferential claims across worlds, and thereby disregard Intrinsic Linkage. Accordingly, it is useful to distinguish between two different types of thought experiments that are commonly employed in debates on personal identity.

A family of thought experiments that I take to be methodologically adequate are what I shall call

Hypotheticals: i.e., thought experiments that are in keeping with the set of facts of the actual world that affect real-life persons and their identity.

Hypotheticals are frequently used in both philosophy and science. By and large, they pose few problems in method, though, as stated by Coleman (2000), “they can certainly cause great disagreement over the results that they may suggest.”

A well-known example of a Hypothetical in personal identity is Thomas Reid’s (1785/1969) Brave Officer, where we are asked to imagine a small boy who once was flogged for having stolen an apple. When that small boy grew up to become a brave officer, he still remembered the flogging. And when the brave officer became an old general, he likewise remembers how he once was a brave officer. However, the old general no longer remembers having been flogged as a small boy. We are then invited to intuit whether the old general is identical to the small boy, despite no longer remembering the flogging. The results yielded by this Hypothetical might be controversial; but its method is not. Brave Officer itself cannot reveal whether the old general is identical to the small boy. It can only serve as a test case for theories of personal identity that have certain implications vis-à-vis the case. These implications are then compared to intuitions pumped by the Hypothetical, and squared with plausible conceptual commitments. Take the memory criterion that Brave Officer is directed against. If continuous first-personal memory is both necessary and sufficient for personal identity, the small boy and the brave officer are identical. As are the brave officer and the old general. But the small boy and the old general are not identical, since there is no first-personal memory relation between the two. This conclusion is not only counterintuitive to most, but also reveals that the memory criterion violates a plausible conceptual commitment about personal identity: if *A* is identical to *B*, and *B* is identical to *C*, then, by transitivity, *A* must also be identical to *C*.

Such analysis of Brave Officer has led many to conclude that the memory criterion of personal identity is implausible. This goes to show that Hypotheticals have a legitimate place in the debate and can help carving out core conceptual conditions of personal identity.

A family of thought experiments that I take to be methodologically inadequate are what I shall call

Counterfactuals:^4^ i.e., thought experiments that are logically possible, but at odds with the set of facts of the actual world that affect real-persons and their identity.

Thought experiments of such nature are particularly widespread in the personal identity literature. Teletransportation, fission and fusion, as well as brain/cerebrum transplants—to name but a few—figure prominently.

One of the most pertinent Counterfactuals is John Locke’s (1698/2012) the Prince and the Cobbler. Locke, intending to establish consciousness as a necessary and sufficient condition of personal identity, asks us to concur that “should the soul of a prince, carrying with it the consciousness of the prince’s past life, enter and inform the body of a cobbler, as soon as deserted by his own soul, everyone sees he would be the same person with the prince, accountable only for the prince’s actions.”

In what follows, I look at a modern-day variant (the naturalization) of the Prince and the Cobbler, and argue why such Counterfactuals, despite appearances, do not yield insights into the ontology of real-life persons.^5^

## Cerebrum transplant counterfactuals

It is widely acknowledged that real-life persons undergo continuous biological changes that are no threat to their diachronic personal identity. The human body’s cells are constantly replaced, and the brain cell connections and chemistry are frequently changing without having an identity-compromising effect; neither on one’s biological nor on one’s psychological make-up. In all ordinary cases, when psychological continuity is in place, so is biological continuity; though not vice versa.^6^

To contrast Psychological Continuity theories with Animalism, it is tempting to imagine what were to happen if psychological continuity were present, but biological continuity were not. Since Locke’s the Prince and the Cobbler with its Cartesian ring of an immaterial soul as the carrier of consciousness is no longer very popular with naturalistically minded philosophers, there has been a shift towards cerebrum transplant Counterfactuals. Accordingly, the soul has been replaced by the cerebrum as the seat of psychological continuity. Cerebrum transplants seem particularly pertinent since they appear to do justice to Naturalism about personhood. That way, cerebrum transplants strike most as less bizarre than, for example, teletransportation or fission Counterfactuals.

Here’s a typical portrayal of a cerebrum transplant: Imagine *A*’s cerebrum is successfully transplanted into *B*’s head, while leaving *A*’s brainstem and midbrain regions intact such that *A*’s organism remains alive. Imagine further that this makes the resulting *B* psychologically continuous with *A* before the transplant had occurred by any standard: *A*’s mental states are physically realized throughout the process, and there are no troublesome rival candidates (Olson 2016). Now, who wakes up after the procedure? The seemingly natural intuition is that, were such things to happen, person *A* would be transferred with their cerebrum. Call this Transplant Intuition. Shoemaker (1963) presents, as do many others, such cerebrum transplant Counterfactuals as decisive evidence for Psychological Continuity theories against Animalism. Modern-day Animalists such as Snowdon (2014), however, disagree, denying the force of Transplant Intuition.^7^

When discussing intuitions gathered from possible world modalities, it is vital to keep in mind that the ‘results’, so yielded, are taken to settle differences between rival views of personal identity regarding real-life persons. It’s not quite the claim that only people in some possible world where cerebrum transplants take place are transferred with their cerebrum. The point is, rather, that pondering these Counterfactuals is supposed to reveal that Psychological Continuity is the correct view of personal identity in real life. Granted, for argument’s sake, that the transplant intuition offers enough of a compelling reason to drop Animalism. We are not, then, identical to the living organism left behind in a cerebrum transplant. Rather, we cease to exist once our psychology is gone; at least we are no longer inhabiting that cerebrum-robbed organism (Parfit 2012). Practically, this could mean that, given the serve deterioration of autobiographical memory in Alzheimer’s that comes with a reported loss of sense of identity (El Haj et al. 2017), advance directives regarding someone in the late stages of Alzheimer’s, with little to no psychological continuity linking them to the initial signee, should not be considered authoritative. By the same token, in the absence of advance directives, there is seemingly no point in interviewing close relatives to reconstruct the presumed patient’s will since the patient currently undergoing treatment is no longer identical to the would-be signee.

There are, however, several constraining facts about the actual world that preclude cerebrum transplants from ever happening.^8^ For one, the underlying assumption that the cerebrum *alone* maintains psychological continuity is called into question by evidence from cognitive science. Theories of embodied cognition (Clark 1997, 1999; Lakoff and Johnson 1999) highlight the interdependence of brain and body. Roughly, the cognitive science of embodied cognition holds that a person’s mind is deeply dependent upon their bodily features. That is, aspects of a person’s body beyond the brain play a significant causal or physically constitutive role in cognitive processing (Wilson et al. 2021). Even if one were able to successfully transplant an entire functioning brain (let alone just the cerebrum), the psychological make-up of the resulting person would be shaped and informed by the constitution of an altogether different body. Granting that the old body and the new were much alike, they’d inevitably still be ever so slightly different, and so would be the resulting person’s psychological make-up. Schechtman (1997) has called this the ‘Brain–body Problem’ and presented an alternative ‘Distributed View’ of the mind which coheres well with evidence from cognitive science. A further line of empirical research suggests that there is a strong ‘brain–body historicity’ based on immunological mechanisms observed in brain tissue transplantations. The immune system distinguishes the body’s own tissue from foreign tissue only on the basis of the quality of the inserted material, whereas the quantity of inserted material is largely irrelevant. Even if the quantity of foreign inserted material is small, the immune system may still reject it. Thus, from an immunological perspective, there appear to be no principled differences between brain tissue transplantations and entire cerebrum transplantations: both are subject to the close interdependence between brain and body (Munzer 1994). We cannot expect, therefore, to transplant a cerebrum into someone else’s head, assuming that this would result in the original person’s distinct psychology having been transplanted. Rather, the entire body’s vital functions, including, but not limited to, the functioning cerebrum, are necessary to sustain a person’s distinct psychological make-up—suggesting that psychological continuity supervenes upon biological continuity.

Psychological continuity—qua being constrained by contingent empirical facts about the human body’s nature—coincides via nomological necessity with biological continuity. Imagining apart psychological continuity from biological continuity, as we are asked to do in cerebrum transplant Counterfactuals, so as to isolate psychological continuity as the dependent variable, and to substitute biological continuity with independent variables (or different causes of psychological continuity), violates the nomologically necessary interdependence of psychological and biological continuity. If psychological continuity supervenes upon biological continuity in all actual cases, the mere conceptual possibility of them coming apart can’t serve as a valid source of intuition when it comes to puzzles about real-life persons.

Despite appearances, Transplant Intuition is not just unreliable when employed to inform judgments about personal identity in the actual world, but largely irrelevant. For, in stepping into the counterfactual perspective, we are intuiting about beings whose envisioned physiological constitution is decisively different from real-life persons, such that reapplying these intuitions back to the actual world constitutes a change of subject.

In the succeeding section, I abstract from cerebrum transplant Counterfactuals to argue, more generally, that intuitions about personal identity gathered from possible worlds that differ in their facts from the actual world to the point of conceptual incongruence, are inadequate when reapplied to real-life persons.

## Intuiting across conceptually incongruent worlds

Counterfactuals consult modalities about possible worlds where things are (often strikingly) different from how they are in the actual world. Typically, physical constraints that preclude, say, fission or teletransportation from actually happening, are imagined away. The implicit assumption seems to be, then, that the concept of personal identity is insensitive to constraining facts of the actual world such that personal identity can be isolated, transferred to some possible world, tested in those possible conditions to finally reapply the intuitions so gathered by transferring them back to the actual world. In so doing, the gathered intuitions from possible worlds are applied to hypothetical cases to see whether the theoretical implications that were drawn out by counterfactual thought-experimenting appear intuitively plausible. If these theoretical implications do not appear plausible in light of the counterfactual, conceptual engineering is undertaken to adjust the theory of personal identity accordingly.^9^

Table 1 compares a possible world *P* where, by stipulation, the set of facts that constrain personal identity is different from the set of facts that constrain personal identity in the actual world *Q*. Say X_1_ about *P* allows for *insert your favorite Counterfactual*; whereas Y_1_ about *Q* precludes said Counterfactual. We have made it true, by stipulation, that people inhabiting *P* survive (or, as the case may be, do not survive) changes enabled by X_1_ that people in *Q*, because of Y_1_, never face. It is hard to see how intuiting about whether people in *Q* would survive a scenario that never occurs^10^ can, in principle, have a sensible—let alone accurate—answer. Since such things never happen to people in *Q*, the conceptual apparatus that has evolved in conjunction with facts about *Q* is ill-equipped to deal with such Counterfactuals. Employing intuitions gathered from pondering what happens to people in *P* to then make inferences about personal identity in *Q* is invalid because of factual incongruences between worlds. Thus, it is unsurprising that pondering about what happens to people in *P* where, say, teletransportation is possible, evokes intuitions that are invalid when reapplied to real-life persons inhabiting *Q*. Such Counterfactuals throw a spanner in the works, then, by leading us to question whether the concept of personal identity applies to these sorts of Counterfactuals, where, in fact, it does not.

**Table 1:** Constraining facts: possible world vs. actual world

Possible world *P*	Actual world *Q*
Personal identity obtains iff {X_1_, X_2_, …, X_n_}	Personal identity obtains iff {Y_1_, Y_2_, …, Y_n_}
Where {X_1_, X_2_, …, X_n_} is the set of constraining facts about personal identity in *P*	Where {Y_1_, Y_2_, …, Y_n_} is the set of constraining facts about personal identity in *Q*

Recall that according to Intrinsic Linkage there is an intimate relation between personhood and relevant constraining facts about the actual world. Had *Q* been different, such that X_1_ would not obtain but Y_1_ would obtain, the concept of personhood, too, would have evolved differently. Intrinsic Linkage suggests, then, that factual incongruences between {X_1_, X_2_, …, X_n_} and {Y_1_, Y_2_, …, Y_n_} imply conceptual incongruences between personhood in *P* and personhood in *Q* that renders intuiting across worlds invalid. What decides the validity of a thought experiment about personal identity is thus the question as to whether the ‘intuition-inference’ from *P* to *Q* implicitly attempts to carry over facts about *P* to *Q* that are incongruent. If so, intuiting across worlds is invalid.

One might object that, since we have historically been mistaken about facts of the actual world, and have based our concept of personal identity on these alleged facts that turned out to be erroneous, Intrinsic Linkage might not be as tight after all. For example, during the heyday of Dualism, it seemed plausible that personal identity is to be analyzed in terms of the persistence of an immaterial soul. With growing knowledge about the actual world, though, we came to realize that the existence of an immaterial soul is rather unlikely. Accordingly, the concept of personal identity has been adjusted, and the soul theory has largely been abandoned.

Rather than viewing our epistemic limitations as an objection to Intrinsic Linkage, our conceptual responsiveness to relevant facts about the actual world indicates that there is an intimate relation between personhood and these constraining facts. We are, and ought to be, prepared to revise our concept of personhood, given newly acquired evidence. In this spirit, Bakhurst (2005) contends, “the marks of personhood issue from facts about what we are, so that there can be weighty truths, presently obscure to us, the discovery of which would dictate how we should think of ourselves.” Furthermore, our *epistemic limitations* that suggest an epistemologically contingent relation between personal identity and what we currently know about the actual world do not rule out an *ontological dependency* between personal identity and relevant constraining facts about the actual world. The correct theory of personal identity cannot be divorced from those facts, but must be responsive to them. When imagining away the actual world’s constraints on persons and their identity, the conceptual boundaries of personal identity dissolve with them. At the very least, the concept of personal identity becomes so blurry that it no longer evokes reliable intuitions—﻿let alone informing sensible judgments about real-life persons.

I now turn to look at how invalid intuition-inferences might be based on conflating de re necessity and de re possibility about persons, and how such a conflation is at odds with Orthodox Commitments. That is, what might constitute personhood in some possible world carries no weight on the whereabouts of real-life persons.

## Violating orthodox commitments

To see how the common practice of invalidly intuiting across worlds might be connected to conflating different de re modalities about persons, it is useful to draw a distinction between de re necessity and de re possibility.

If, via Counterfactuals, we are to isolate what is conceptually essential about persons and their identity per se, the features that constitute personhood must be steady across worlds. Call this de re necessity about personhood, according to which,

in every possible world containing persons, persons are F; whereby F contains every constitutive feature all and only persons possess necessarily.

As per Cognitivism, one such feature is higher-order cognition. However, there are logically possible worlds where persons are just like us; except, they have no psychological features at all (akin to Chalmers’s Zombies). Such Zombie-like persons might still employ the de dicto practice of successfully ascribing personhood to each other in their respective possible world. Zombie-like persons might, for example, be held morally accountable for their actions; not based on any actual mental life though, but solely based on their behavior. Cognitivism, then, is not steady across worlds. It is, however, a widely agreed upon feature of real-life persons that few philosophers are willing to drop.

If, via Counterfactuals, we take the more moderate aim to isolate what is conceptually essential about persons and their identity per alia, the features that constitute personhood must be steady only within worlds. Call this de re possibility about personhood according to which,

in at least one possible world containing persons, persons are G; whereby G contains every constitutive feature all and only persons possess necessarily in that particular world.

This might well be true; however, we cannot expect de re possibility to enable valid inferences across worlds. Zombie-like persons (lacking higher-order cognition, and any sort of consciousness, for that matter), for example, might very well count as persons in some possible world. But that does not yield any insights into real-life persons that are constituted differently (possessing higher-order cognition that enables them to track themselves over time).

If Cognitivism is true, persons are equipped with higher-order cognition that supervenes upon biological facts about their brains and bodies. Personal identity can, then, only be analyzed properly by being responsive to biological facts about higher-order cognition.

Furthermore, both de re necessity and de re possibility about personhood are in tension with Realism. If ‘person’ is a natural kind, persons cannot exist outside of their natural habitat. We cannot, it seems, have it both ways: holding on to Realism and conceptually removing persons from the actual world, placing them in some possible world. There is a related problem with holding on to Naturalism: if persons are biological beings whose existence is a matter of empirical facts, it is conceptually erroneous to disregard these empirical facts when considering Counterfactuals, and, simultaneously expect inferences drawn from pondering such scenarios to be informative regarding real-life persons.

Intuiting from Counterfactuals to make claims about real-life persons and their identity, both in terms of de re necessity and de re possibility, thus requires dropping any number of Orthodox Commitments. Biting the bullet, though, comes at a high price that, presumably, few philosophers are prepared to pay.

Having tidied up some of the conceptual muddle, I now turn to shed light on a few promising strategies that have recently been put forward to deal with troublesome implications of theoretical convictions in personal identity derived from counterfactuals when it comes to real-life cases.

## Theoretical convictions, empirical studies, and real-life applications

The previously mentioned studies of folk intuitions regarding personal identity (footnote 5) point to the recent rise of experimental methods in philosophical discussions on personal identity. These empirically-informed approaches are an important step towards challenging the weight accorded to armchair theorizing in discussions of healthcare and health policy issues related to personal identity.^11^

In an online survey, Strohminger and Nichols (2014) investigated—employing case-study experiments (including a version of the brain transplant)—that moral traits, rather than other cognitive functions, are perceived to be the most integral part of personal identity. Contrary to theoretical convictions of psychological continuity views that do not properly account for the importance of differences in psychological traits in preserving personal identity, these findings suggest that folk notions of personal identity are largely informed by what the authors call the ‘essential moral self’, according to which the mental faculties affecting social relationships, with a particularly keen focus on moral traits, are most relevant to personal identity.

In a follow-up study, Strohminger and Nichols (2015) studied changes in personal identity in patients with various kinds of neurodegenerative diseases (dementia, Alzheimer’s, and amyotrophic lateral sclerosis) as perceived by patients’ relatives. Participants were told that the purpose of the research was to investigate how the neurodegenerative disease affected personal relationships. Accordingly, participants were asked questions indicative of how much the patient had changed since the onset of the disease. The study’s results suggest that damage to the moral faculty is particularly threatening to the personal identity of patients as perceived by relatives. While other cognitive deficits did not show measurable impact on personal identity. Neurodegenerative diseases such as frontotemporal dementia that attack the brain’s moral processing faculty have shown the greatest effect on perceived change in personal identity; whereas neurodegenerative diseases such as amyotrophic lateral sclerosis that affect mostly cognitive processing have shown the least effect on perceived change in personal identity. Needless to say, the presumed personal identity of patients as third-personally perceived by relatives cannot simply be converted into what patients themselves experience first-personally, and so there are limits to what we can learn from these results. But if these studies are at all indicative of what people want for themselves in terms of medical treatment, the results should be taken seriously into account when it comes to advance directives.

More generally, these kind of empirical studies have a potentially important impact on theorizing about personal identity since they call into question widespread views according to which personal identity consists mainly in unspecified psychological continuity. At the very least, these results indicate that a more fine-grained theoretical analysis of just which features of psychological continuity in detail are identity-preserving is required; suggesting that the coarse-grained concept of psychological continuity is not equipped with the necessary conceptual sophistication needed to inform real-life decisions about personal identity. Furthermore, if taken at face value, the ‘essential moral self’ view has serious ramifications for personal identity in light of moral enhancement. If moral traits are essential to personal identity, altering one’s moral traits via, say, pharmacological intervention could, in principle, change a person to the point of becoming a different person altogether. Crutchfield (2018) goes so far as to suggest that moral enhancement can ‘kill’ the enhanced person.

With regard to the potential threat that DBS poses to personal identity, Bluhm et al. (2020) have recently made the case for utilizing empirical data gathered from patient reports both for improving patient care, and informing theories of personal identity. Their findings suggest the actual experiences of patients having undergone DBS cohere much more with a relational understanding of personal identity, according to which personal identity is formed within a web of social relations (Schechtman 2014), than with metaphysical reductionism about personal identity, where persons can be entirely reduced to the existence of certain psychological and/or biological states and their various relations (Parfit 1984). Bluhm et al. (2020) report, for example, that the majority of people that undergo DBS do not feel that they have changed in any fundamental way after DBS; at least not more so than the alterations they have experienced as a result of their illness, or of pharmacological treatments. Taking these patient narratives seriously, then, leads to a more nuanced reading of these reports that may have concrete, practical and theoretical implications. Contextualizing actual patients’ experiences calls for asking ‘what is it like to be a person being treated with DBS’ rather than asking ‘whether DBS is a threat to personal identity’. This is not just a semantic sleight of hand, but might contribute to a better understanding of what actually happens to DBS patients’ personal identity, and thus help create tailor-made health care policies.

## Concluding remarks

I have argued against the adequacy of employing counterfactual thought experiments that are at odds with relevant facts about the actual world to make claims about real-life persons and their identity. Personhood is deeply rooted in the actual world’s constraining facts such that persons can’t be conceptually isolated from the inner workings of the world they inhabit, without changing the concept fundamentally. In so doing, we are not talking about the identity of persons *as we know them* anymore, but about the identity of imaginary persons* instead. What makes persons* persist, however, carries no weight for real people.

If my arguments are on the right track, the onus lies with proponents of Counterfactuals to demonstrate the need and adequacy of reverting to such scenarios. Rather than pondering bizarre Counterfactuals, it might be worthwhile taking more seriously real-life puzzles that are far from solved. What happens to the identity of persons suffering from disorders of consciousness (such as persistent vegetative state) or neurodegenerative diseases (such as late stages of Alzheimer’s), for example, is extensively discussed in the medical ethics literature of advance directives. Given the ontological dependency between personhood and the relevant actual world’s constraining facts, these and other conditions might deserve more theoretical attention than they currently garner. Resulting empirically-informed theories of personal identity will be both ontologically more plausible, and better able to shed light on novel clinical applications that potentially alter real people’s identities.

## Footnotes

1 Frankfurt (1971), Dennett (1976), Baker (2000), Wiggins (2001), McMahan (2003), Olson (2007), Shoemaker (2008), and Parfit (2012) are among the most prominent advocates of Orthodox Commitments.

2 Parfit understands that some thought experiments will remain forever impossible. Whereas others are merely technologically impossible or, in fact, possible. I return to these distinctions shortly.

3 Wilkes (1988), Gendler (1998, 2002), and Kipper (2016) are wary of employing counterfactual thought experiments in personal identity. Coleman (2000), and Beck (2006; 2016), on the other hand, defend their adequacy.

4 I use the terms Hypotheticals and Counterfactuals exclusively to denote factually possible and factually impossible thought experiments with regard to persons and their identity, respectively. So, when I say counterfactual, I do not talk about conditionals that make claims about circumstances that would have followed, had the actual sequence of events been different.

5 A recent approach in experimental philosophy has been to study the robustness of folk intuitions regarding thought experiments in personal identity (Blok et al. 2005; Nichols and Bruno 2010; Berniūnas and Dranseika 2016). These studies do not, however, aim at defending the role of intuitions in assessing theories of personal identity. Rather, the goal is to show “that if it is appropriate for philosophers to rely on intuitions in assessing theories of personal identity, then it will help to identify which intuitions are especially robust (Nichols and Bruno 2010)”. I am not arguing against studying folk intuitions about personal identity, neither regarding counterfactual nor hypothetical cases. What I am concerned with is the counterfactual method in itself; i.e., employing intuitions pumped by thought experiments that are at odds with the relevant facts of the actual world about persons and their identity to inform the de facto ontological make-up of real people.

6 Persistent vegetative state is an obvious example where biological continuity obtains but psychological continuity has vanished.

7 For a recent analysis of transplant intuitions in the debate on the metaphysical soundness of Animalism see Skrzypek and Mangino (2021).

8 An anonymous referee pointed out that the empirical constraints not so much preclude cerebrum transplants from happening, but call into question the claim that such transplants are sufficient (or, indeed, necessary) to preserve personal identity when psychological and biological continuity come apart.

9 I am grateful to an anonymous referee for having me flesh out the idea of ‘intuition-transfer’.

10 By an event ‘never occurring’ I mean that the event is impossible to occur due to the actual world’s constraining facts—not just that the event has not yet occurred. An anonymous referee has rightly pointed to the epistemic limitations that we face in figuring out whether an event is impossible to occur necessarily or whether it just so happens that it hasn’t occurred yet. Given the epistemic uncertainty regarding the future state of science and technology, I do not mean to suggest that counterfactually generated philosophical intuitions should not be accorded any role at all in theorizing about personal identity. For example, counterfactual thought experiments can be useful to carve out differences between rival views that appear less strikingly so in ordinary cases.

11 I am grateful to an anonymous referee for urging me to discuss the important experimental work on personal identity.
